# Treatment rationale and study design for a phase III, double-blind, placebo-controlled study of maintenance pemetrexed plus best supportive care versus best supportive care immediately following induction treatment with pemetrexed plus cisplatin for advanced nonsquamous non-small cell lung cancer

**DOI:** 10.1186/1471-2407-10-85

**Published:** 2010-03-08

**Authors:** Luis G Paz-Ares, Sedat Altug, Alexandra Thareau Vaury, Jesús Corral Jaime, Francesca Russo, Carla Visseren-Grul

**Affiliations:** 1 University Hospital - Virgen del Rocio, Seville, Spain; 2 Eli Lilly and Company, Istanbul, Turkey; 3 Eli Lilly and Company, Paris, France; 4 Eli Lilly Italy, Florence, Italy; 5 Eli Lilly and Company, Houten, The Netherlands

## Abstract

**Background:**

To improve the efficacy of first-line therapy for advanced non-small cell lung cancer (NSCLC), additional maintenance chemotherapy may be given after initial induction chemotherapy in patients who did not progress during the initial treatment, rather than waiting for disease progression to administer second-line treatment. Maintenance therapy may consist of an agent that either was or was not present in the induction regimen. The antifolate pemetrexed is efficacious in combination with cisplatin for first-line treatment of advanced NSCLC and has shown efficacy as a maintenance agent in studies in which it was not included in the induction regimen. We designed a phase III study to determine if pemetrexed maintenance therapy improves progression-free survival (PFS) and overall survival (OS) after cisplatin/pemetrexed induction therapy in patients with advanced nonsquamous NSCLC. Furthermore, since evidence suggests expression levels of thymidylate synthase, the primary target of pemetrexed, may be associated with responsiveness to pemetrexed, translational research will address whether thymidylate synthase expression correlates with efficacy outcomes of pemetrexed.

**Methods/Design:**

Approximately 900 patients will receive four cycles of induction chemotherapy consisting of pemetrexed (500 mg/m^2^) and cisplatin (75 mg/m^2^) on day 1 of a 21-day cycle. Patients with an Eastern Cooperative Oncology Group performance status of 0 or 1 who have not progressed during induction therapy will randomly receive (in a 2:1 ratio) one of two double-blind maintenance regimens: pemetrexed (500 mg/m^2 ^on day 1 of a 21-day cycle) plus best supportive care (BSC) or placebo plus BSC. The primary objective is to compare PFS between treatment arms. Secondary objectives include a fully powered analysis of OS, objective tumor response rate, patient-reported outcomes, resource utilization, and toxicity. Tumor specimens for translational research will be obtained from consenting patients before induction treatment, with a second biopsy performed in eligible patients following the induction phase.

**Discussion:**

Although using a drug as maintenance therapy that was not used in the induction regimen exposes patients to an agent with a different mechanism of action, evidence suggests that continued use of an agent present in the induction regimen as maintenance therapy enables the identification of patients most likely to benefit from maintenance treatment.

**Trial Registration:**

**Trial Registry**: Clinicaltrials.gov

**Registration number**: NCT00789373

**Trial abbreviation**: H3E-EW-S124

## Background

More than 75% of patients with non-small cell lung cancer (NSCLC) present with locally advanced (stage IIIB) or metastatic (stage IV) disease [1]. Treatment for these patients includes chemotherapy, radiotherapy, and best supportive care (BSC), with current guidelines recommending platinum-based combination regimens as first-line treatment [2,3]. Although improvement has been achieved over the last few decades, the prognosis for these patients remains poor, with response rates for first-line therapy of 20% to 40% and median survival times of 7 to 12 months [3,4].

Numerous efforts have been made to improve the efficacy of first-line therapy for advanced NSCLC. Although differing in tolerability, standard platinum-based doublets have not appeared to differ in efficacy. For example, in a randomized phase III study comparing cisplatin/gemcitabine, cisplatin/paclitaxel, cisplatin/docetaxel, and carboplatin/paclitaxel, all of the regimens demonstrated comparable median overall survival (OS) [5]. Likewise, in another study comparing the first two of the previous doublets to cisplatin/vinorelbine, a superior regimen was not identified [6]. Various non-platinum-containing regimens have also been tested, but none have been proven superior [3]. Additionally, prolonged first-line treatment does not appear beneficial [7-10].

Molecular targeted therapy has recently been examined as a means to improve the efficacy of advanced NSCLC first-line therapy. The epidermal growth factor receptor (EGFR) tyrosine kinase inhibitors erlotinib and gefitinib did not improve efficacy when added to a platinum doublet [11]. They were active, however, as single agents in the second- and third-line setting, particularly in patients with EGFR mutations [11,12]. Cetuximab and bevacizumab, monoclonal antibodies against the EGFR and the vascular endothelial growth factor, respectively, resulted in improved survival when added to standard first-line therapy [13-16]. However, cetuximab is not currently approved for NSCLC treatment, and toxicity limits the use of bevacizumab to nonsquamous tumors [17]; therefore, platinum-based chemotherapy doublets remain the treatment of choice for many patients.

Another means of optimizing first-line therapy has been to examine whether superior treatment outcome is associated with specific patient or disease characteristics. In a phase III study in patients with advanced NSCLC, a preplanned subgroup analysis showed a difference in response by tumor histology [18,19]. Patients with nonsquamous histology (n = 1000) had statistically superior OS with cisplatin/pemetrexed than cisplatin/gemcitabine (11.0 versus 10.1 months; hazard ratio [HR] = 0.844, p = 0.011) [19]. A retrospective analysis of previous pemetrexed phase III trials confirmed the favorable efficacy seen with pemetrexed in nonsquamous NSCLC [19]. The differential responsiveness seen may be associated with differences in thymidylate synthase (TS), the primary target of the antifolate pemetrexed [20,21]. TS levels are approximately 1.5-fold lower in adenocarcinoma compared with squamous cell carcinoma [22,23]. Preclinical evidence suggests that high levels of TS may indicate lowered sensitivity to antifolates [24,25].

In addition to the identification of novel agents and combinations for first-line treatment of advanced NSCLC, efforts have focused on improving efficacy and tolerability via alterations in the treatment schedule [26-28]. In maintenance therapy (also referred to as consolidation therapy), the duration of chemotherapy is prolonged by administering additional drugs in patients who have not progressed or who have achieved a response during the initial or induction chemotherapy. It is administered for either a defined number of cycles or until progressive disease. The goals of maintenance therapy are to improve OS, slow disease progression, and maintain or improve the quality of life without untoward side effects.

Post-induction maintenance therapy has the theoretical advantage of allowing patients to continue chemotherapy while the tumor burden is low (compared to treatment after disease progression). By using a different drug for maintenance therapy than that used in the induction regimen, patients are exposed to a chemotherapeutic agent with a different mechanism of action. Alternatively, using the same drug for maintenance therapy that was effective during the induction regimen offers the practical advantage of continuing a beneficial chemotherapy with the favorable toxicity profile of a single-agent treatment. In fact, Grossi and colleagues [27] have likened the induction phase to an in-vivo drug-sensitivity assay because it selects for patients who achieve a minimum response to the induction therapy (either response or stable disease), and who are thus more likely to benefit from maintenance chemotherapy with one of the drugs included in the induction regimen.

One of the first phase III studies that explored maintenance therapy examined gemcitabine maintenance therapy following four cycles of induction therapy with cisplatin/gemcitabine [29]. Patients without progressive disease following induction therapy randomly received either gemcitabine or BSC. The median time to progressive disease was significantly longer in patients who received gemcitabine maintenance therapy versus BSC. Additionally, a significantly longer OS time occurred in patients with a high baseline Karnofsky performance status (PS) (>80) who were treated with gemcitabine compared with BSC, whereas the difference in survival was not statistically significant between arms in patients with a low baseline performance status. A recent phase III study compared docetaxel given either immediately after 4 cycles of carboplatin/gemcitabine (as a maintenance therapy) or after disease progression (as a second-line therapy) [30]. A statistically significant improvement in progression-free survival (PFS) and a numerical increase in OS were observed in patients who received docetaxel as maintenance therapy.

Because of the efficacy of pemetrexed in second-line NSCLC [31], its favorable safety profile, and its ease of administration (a 10-minute infusion), a phase III study examined pemetrexed (versus placebo) as maintenance therapy in patients who had not progressed following administration of one of six non-pemetrexed-containing, commonly prescribed induction regimens: carboplatin/gemcitabine, cisplatin/gemcitabine, carboplatin/docetaxel, cisplatin/docetaxel, carboplatin/paclitaxel, or cisplatin/paclitaxel [32,33]. Pemetrexed maintenance therapy resulted in a statistically significant and clinically relevant increase in OS and PFS in the overall and nonsquamous populations [33]. The improved efficacy observed in patients with nonsquamous tumors confirmed previous findings for pemetrexed in NSCLC [18,19]. The study also demonstrated that pemetrexed maintenance therapy was well tolerated [34].

Given the efficacy of pemetrexed in combination with cisplatin as a first-line doublet and as a maintenance agent (when not used in the induction regimen), we designed a phase III study to determine if pemetrexed maintenance therapy will improve efficacy over placebo following cisplatin/pemetrexed induction therapy in patients with advanced nonsquamous NSCLC. Furthermore, translational research will address whether TS expression correlates with efficacy outcomes in patients treated with pemetrexed.

## Methods

### Objectives

The primary objective of this study is to compare PFS of pemetrexed maintenance therapy plus BSC versus placebo plus BSC in patients with stage IIIB or IV nonsquamous NSCLC whose disease has not progressed during four cycles of pemetrexed and cisplatin induction chemotherapy.

Secondary objectives of the study are to compare the following between treatment arms: OS; objective tumor response rate (assessed using Response Evaluation Criteria in Solid Tumors [RECIST] [35]); patient-reported outcomes (using the EuroQol 5-dimensional scale [EQ-5D] [36]); resource utilization; and toxicity (adverse events graded using Common Terminology Criteria for Adverse Events [CTCAE] [Version 3.0] [37]). The objective of the translational research component of the study is to examine the association between TS expression and efficacy outcomes.

### Study Design and Treatment Plan

**Figure 1:**
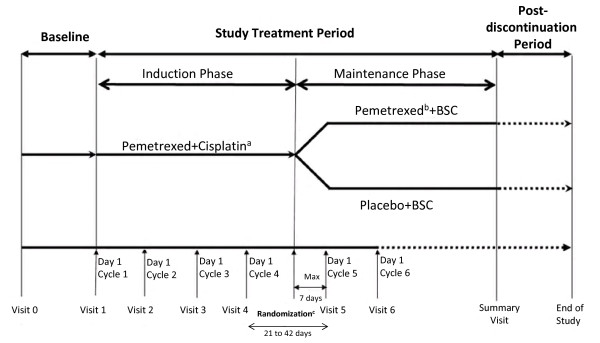
**Study Design**. Abbreviations: BSC = best supportive care. aPemetrexed (500 mg/m2, day 1) plus cisplatin (75 mg/m2, day 1). bPemetrexed (500 mg/m2, day 1). cAfter completion of 4 cycles of induction treatment, eligible patients without disease progression will be randomized 2:1 to receive pemetrexed plus BSC or placebo plus BSC during the double-blind maintenance phase of the study.

The study will have two treatment phases: an unblinded induction phase and a blinded maintenance phase (Figure 1). In the induction phase, eligible patients will receive four cycles of induction chemotherapy consisting of pemetrexed (500 mg/m^2^) and cisplatin (75 mg/m^2^) on day 1 of a 21-day cycle.

Following completion of cycle 4 of induction treatment, eligible patients will be randomly assigned to one of two double-blind maintenance regimens: pemetrexed 500 mg/m^2^, administered intravenously on day 1 every 21 days, plus BSC (pemetrexed arm); or approximately 100 mL normal saline (0.9% sodium chloride), administered intravenously on day 1 every 21 days, plus BSC (placebo arm). Maintenance chemotherapy will begin at the time of randomization or a maximum of 7 days from the date of randomization. Patients must start maintenance therapy no earlier than 21 days and no later than 42 days from day 1 of the fourth cycle of induction therapy. Randomization to treatment arm will be stratified by the following prognostic factors: Eastern Cooperative Oncology Group (ECOG) PS just prior to randomization (0 versus 1), tumor response to induction chemotherapy (complete/partial response [CR/PR] versus stable disease [SD]), and disease stage prior to administration of induction therapy (IIIB versus IV). Maintenance therapy will end when the patient meets one of the prespecified reasons for discontinuation, including disease progression. Patients who discontinue maintenance treatment without progressive disease will continue to receive periodic tumor response evaluation. Once a patient has progressed, follow up will occur every 90 days until death or study closure. Information regarding anticancer systemic therapy, radiotherapy, and surgical intervention will be collected during the post-discontinuation period.

During the study, all patients will receive standard folic acid and vitamin B_12 _supplementation and prophylactic dexamethasone, as specified in the pemetrexed label.

The protocol will be approved by each participating institutional ethics review board. The study will be conducted in accordance with the ethical principles of the Declaration of Helsinki and good clinical practice. All patients will sign written informed consent before treatment.

### Study Population

Patients will be eligible for participation in the induction phase of the study if they have: histologic or cytologic diagnosis of advanced (stage IIIB with pleural effusion and/or positive supraclavicular lymph nodes or stage IV) nonsquamous NSCLC; no prior systemic chemotherapy for lung cancer; ≥1 measurable lesion; an ECOG PS of 0 or 1; adequate organ function; and ≥18 years of age. Patients will be ineligible for the study if they have predominantly squamous cell and/or mixed small cell, non-small cell histology, or are receiving concurrent administration of any other antitumor therapy. Patients will be eligible for randomization and treatment in the maintenance phase of the study if they: complete four cycles of induction therapy; have documented (confirmed or unconfirmed) radiographic evidence of CR, PR, or SD after completion of induction chemotherapy; and have an ECOG PS of 0 or 1. Table 1 lists the eligibility criteria for both phases of the study.

**Table 1:** Key Patient Selection Criteria

Induction Phase	
*Inclusion Criteria*	*Exclusion Criteria*
• Histologic or cytologic diagnosis of advanced, nonsquamous NSCLC • Stage IIIB (with pleural effusion and/or positive supraclavicular lymph nodes) or IV disease prior to induction therapy not amenable to curative therapy • Prior radiation therapy to <25% of the bone marrow (not including whole pelvis radiation) if completed and patient has recovered 30 days before enrollment • ≥1 unidimensionally measurable lesion as defined by RECIST • Males and females ≥18 years of age • Performance status of 0 or 1 on the ECOG scale • Estimated life expectancy of ≥12 weeks • Adequate organ function (bone marrow reserve, renal, hepatic) • Use of an approved contraceptive method by male and female patients with reproductive potential • Negative serum or urine pregnancy test within 7 days before study enrollment for women with childbearing potential • Ability to comply with study and/or follow-up procedures • Signed informed consent document	• Squamous cell and/or mixed small cell, non-small cell histology • Prior systemic chemotherapy for lung cancer, including adjuvant therapy, for any stage of NSCLC • Concurrent administration of any other antitumor therapy • Treatment within last 30 days with a drug that has not received regulatory approval • Prior participation in a study investigating pemetrexed • Serious concomitant systemic disorder that would compromise ability to adhere to the protocol • Serious cardiac condition, such as myocardial infarction, angina, or heart disease • Prior malignancy other than NSCLC, carcinoma in situ of the cervix, or nonmelanoma skin cancer unless malignancy diagnosed and treated ≥5 years previously without recurrence • Central nervous system metastases unless the patient has completed successful local therapy and has been off corticosteroids for ≥4 weeks • Clinically significant third space fluid collection that cannot be controlled by drainage or other procedure • Inability to interrupt aspirin or other nonsteroidal anti-inflammatory drugs, other than an aspirin dose ≤1.3 g per day, for a 5-day period • Inability or unwillingness to take folic acid, vitamin B_12 _supplementation, or corticosteroids

**Maintenance Phase**	

*Inclusion criteria*	
• ECOG PS of 0 or 1 • Documented radiographic evidence of a complete or partial tumor response or stable disease* • Completion of four cycles of induction chemotherapy	

RECIST = Response Evaluation Criteria in Solid Tumors; ECOG PS = Eastern Cooperative Oncology Group performance status.

*Tumor assessment must occur between cycle 4 (day 1) of induction therapy and the date of randomization. This response does not have to be confirmed in order for the patient to be randomized.

### Statistical Analysis Plan

A minimum of 558 qualified patients will be randomly assigned in a 2:1 ratio to maintenance therapy of pemetrexed plus BSC (372 patients) or placebo plus BSC (186 patients) to provide at least 238 PFS events (52% censoring) for the primary efficacy analysis. Assuming a true PFS HR of 0.65, the primary unadjusted log-rank test of PFS will have 90% power to show a statistical difference between arms. In order to randomize at least 558 qualified patients, approximately 900 patients will be treated during the induction phase.

OS will be analyzed at the time of the primary PFS analysis and at the end of the study. Assuming the true OS HR is 0.70, the final unadjusted log-rank test of OS will have 93% power to show a statistical difference between arms provided there are 390 events (30% censoring). To maintain an overall two-sided alpha level of 5% for the analyses of PFS and OS, a statistical gatekeeping and alpha-spending strategy will be applied.

Separate baseline, efficacy, and safety analyses will be done for the induction and maintenance phases of the study. For the induction phase, the population will consist of patients receiving at least one dose of either pemetrexed or cisplatin. For the maintenance phase, all patients who were randomized to a treatment arm will be eligible for baseline, efficacy, and safety analyses. The primary analysis of PFS will compare maintenance pemetrexed to placebo, calculated from the time of randomization following completion of induction treatment to the first date of objectively determined PD or death from any cause.

The Kaplan-Meier method [38] will be used for OS and PFS time-to-event analyses; the Cox proportional hazards model [39] will be used to estimate HRs with assigned treatment as the only covariate. Tumor response rates (PR + CR) and disease control rates (CR + PR + SD) will be compared between randomization arms using the Fisher's exact test. The tumor response to maintenance therapy will use the last radiologic assessment before randomization as baseline. The tumor response to whole treatment (induction and maintenance) will use the radiologic assessment before induction therapy as baseline.

For patient-reported outcomes analyses, all enrolled patients with a baseline and ≥1 EQ-5D survey will be included in the assessment. EQ-5D findings will be summarized for all patients enrolled at baseline, at each cycle of the induction phase, and at each cycle during the maintenance phase by treatment arm. Mean index and visual analog scale scores will be calculated and analyzed using a mixed effect analysis of variance model. Analysis of resource utilization will include all patients treated with study therapy.

### Translational Research

Patients are eligible to participate in this component of the study if they have achieved a CR, PR, or SD during the induction phase of the study, have tissue obtained from a biopsy prior to starting induction treatment, and have provided informed consent for the collection of a second biopsy before beginning maintenance treatment. Both tissue biopsies must be formalin-fixed and paraffin embedded, with tissue blocks preferred over slides. Intratumoral mRNA expression will be measured in a central laboratory using quantitative polymerase chain reaction techniques. A minimum of 100 paired specimens (baseline and second biopsy samples) will be collected to examine if TS expression correlates with efficacy outcomes of pemetrexed. Other lung and pemetrexed biomarkers will be evaluated if sufficient tissue is available.

## Discussion

This trial examines whether pemetrexed maintenance therapy following four cycles of cisplatin/pemetrexed induction therapy provides an efficacy benefit without substantially affecting patient safety. As noted by Grossi and colleagues [27], a study design such as ours enables us to select only those patients responsive to pemetrexed to continue with pemetrexed maintenance treatment. To better understand the molecular mechanism of pemetrexed responsiveness, TS gene expression before and after induction therapy will be measured and correlated with the efficacy results.

Two phase III studies in patients with NSCLC have examined a platinum-based induction doublet followed immediately by maintenance therapy composed of the non-platinum agent of the induction regimen. In one study by Brodowicz and colleagues, gemcitabine maintenance therapy was administered following cisplatin/gemcitabine induction therapy [29]. In a second study, paclitaxel maintenance therapy was administered following carboplatin/paclitaxel induction therapy [40,41]. In both studies, patients who continued to receive the non-platinum agent of the induction regimen as maintenance therapy had better outcomes than patients who received no maintenance therapy.

The efficacy and safety of pemetrexed as a maintenance therapy has been examined in a small study of patients with malignant pleural mesothelioma [42]. Patients received six cycles of pemetrexed-containing induction therapy composed of either carboplatin/pemetrexed or pemetrexed monotherapy (for patients who had prior first-line therapy). Patients receiving pemetrexed maintenance therapy showed promising increases in time to progression and OS. Additionally, pemetrexed maintenance therapy was well tolerated, with no grade 4 toxicity observed, and fatigue and neutropenia as the only grade 3 events occurring in more than one patient (2 each).

The safety of maintenance pemetrexed following induction treatment with pemetrexed/cisplatin will also be assessed in this study. The safety profile of pemetrexed/cisplatin is well established based on results from two phase III studies in advanced NSCLC [18] and malignant pleural mesothelioma [43]. The safety of single-agent pemetrexed administered as maintenance [32,33] and second-line treatment of advanced NSCLC [31] has also been established. In the maintenance study, the safety of long-term pemetrexed exposure was evaluated in patients who received 10 or more cycles of maintenance pemetrexed (N = 98) following induction therapy with a non-pemetrexed-containing regimen. There was no significant difference in drug-related grade 3/4 adverse events for these patients compared with those who received less than 10 cycles [33].

In an effort to improve the effectiveness of first-line cisplatin/pemetrexed therapy for advanced nonsquamous NSCLC, this study examines pemetrexed maintenance therapy immediately following four cycles of induction therapy. Evidence suggests that following a platinum-containing induction doublet with maintenance therapy using the non-platinum agent of the induction regimen enables the identification of patients most likely to benefit from the maintenance treatment.

## Competing interests

SA, ATV, FR, and CV-G are employees of Eli Lilly and Company; CV-G owns stock in the company. LGP-A has received consulting fees from Eli Lilly and Company. JCJ has no competing interests.

## Authors' contributions

LGPA, SA, ATW, CVG made substantial contributions to the design of the study. All authors contributed to the implementation of the study, were involved in revising the manuscript critically, and gave their final approval of the version to be published.

## Pre-publication history

The pre-publication history for this paper can be accessed here:

http://www.biomedcentral.com/1471-2407/10/85/prepub
